# Supplementary material for: Prioritized Shortest Path Computation Mechanism (PSPCM) for wireless sensor networks
Source: PLoS One. 2022 Mar 10;17(3):e0264683. doi: 10.1371/journal.pone.0264683 (PMC8912138; doi:10.1371/journal.pone.0264683)
Supplement: Attachment — Submitted filename: Responses to Reviewers - Rebuttal Letter.docx [file pone.0264683.s002.docx]

**Innocent Uzougbo ONWUEGBUZIE**
School of Computing, Faculty of Engineering,

Universiti Teknologi Malaysia (UTM)
Johor Bahru, Johor, Malaysia

31th March 2021.

The Editor,

*PLoS ONE*

Dear Editor,

**Rebuttal Letter**

Thank you for inviting us to submit a revised draft of our manuscript entitled, "***Prioritized shortest path computation mechanism (PSPCM) for wireless sensor networks***" for consideration by *PLoS ONE*. We also appreciate the time and effort you and each of the reviewers have dedicated to providing insightful feedback on ways to strengthen our paper. Thus, it is with great pleasure that we resubmit our revised paper for further consideration. We have incorporated changes that reflect the detailed suggestions you have meticulously provided. We also hope that our updates/improvements and the responses we provide below satisfactorily address all the issues and concerns you and the reviewers have raised.

For clarity, we have presented our responses to your comments and questions in a tabular format; *Table 1: Editors’ comment and response, Table 2: Response to the evaluation by Reviewer 1*, and *Table 3: Response to the evaluation by Reviewer 2.*

**Table 1: Editors’ comment and response**

|  | **Editors’ Comments** | **Responses** |
| --- | --- | --- |
|  | Please ensure that your manuscript meets PLOS ONE's style requirements, including those for file naming. | Thank you for your comments.  The paper has been formatted to meet PLOS ONE’s style requirements. |

**Table 2: Response to the evaluation by Reviewer 1**

|  | **Reviewers’ Comments** | **Responses** |
| --- | --- | --- |
| 1 | Overall, the results seem to be interesting and useful. However, a more impactful work would be to understand the type of data and to see if some form of reduction in actual data bits sent, while maintaining full functionality, would conserve more energy. | Thank you so much for your insightful comments and suggestions to improve the quality of our work.  The type of data traversed across the network is in bits and bytes (8 bits equals a byte). By standard, the IEEE 802.15.4 allows for a maximum transmission unit (MTU) of 127 bytes; 25 bytes for MAC Frame Header, and 102 bytes for payload. However, of the 102 bytes for payload, 21 bytes are used for link-layer security, 40 bytes for IPv6 header, and 8 bytes for UDP header. Eventually, the payload is left with only 33 bytes. Depending on the size of data sensed from the monitored environment, the 33 bytes may not be enough. To increase the payload size, the techniques of Header Compression (HC) were used to compress the IPv6 header and UDP header from 48 bytes to 6 bytes. This eventually increased the payload capacity to 75 bytes. For this research, a payload size of 40 bytes was used. This was sufficient enough to ensure the optimal performance of the proposed PSPCM with conserved energy utilization. Further details of this are presented in the third and fourth paragraphs of Section 3.1.  The entire simulation/experimentation was reperformed with the updated parameters. To also show support for PSPCM network scalability, the number of nodes for HPD and LPD comparison was increased from 100 to 300, while the number of nodes for comparison with related mechanisms (i.e. ETX, QCOF, QWL-RPL, RPL-EC, RPL-FL), was increased from 50 to 200. The new findings are discussed in Sections 5.1 and 5.2 |
| 2. | The paper is well organized; however, careful reading is needed to ensure all English-type errors are corrected. | Thank you for your comments.  The entire paper has been proofread, and all grammatical and spelling errors have been corrected. |

**Table 2: Response to the evaluation by Reviewer 2.**

|  | **Reviewers’ Comments** | **Responses** |
| --- | --- | --- |
| 1. | Results for Throughput (Sec. 5.1.3; Fig. 11) only shows performance within the proposed PSPCM (i.e. HPD vs LPD). These must also be compared with other techniques (i.e. ETX, QCOF, QWL-RPL, RPL-EC, RPL-FL) without priority to show that the proposed technique is superior or similar in performance. | Thank you very much for your comments.  The Throughput performance of PSPCM in comparison with related mechanisms (i.e. ETX, QCOF, QWL-RPL, RPL-EC, RPL-FL) is now presented and supported with updated discussion in Section 5.2.3. The result shows that PSPCM outperformed compared mechanisms. |
| 2. | The proposed scheme uses binary values of HPD and LPD. It is not clear what criteria will be used by the source to decide on this binary decision. Greedy sources might set all their packages to HPD, as there is no objective measure or criteria, which will result in a loss of advantages of the proposed scheme. | Thank you for your comments.  The *deciding factor* is the Class of Service (CS) tag of the data, which is pre-configured into the sensor nodes (High Priority Node – HPN and Low Priority Node – LPN) before network operations begin. See Fig. 4. The 8-bits *Reserved* field of the DIO base object which runs in the network layer of the WSN protocol stack of all nodes is modified to accommodate data of varying priorities and this is represented by the CS tags, where bits 00000000 is for CS = 0 representing Low Priority Data (LPD) and bits 00000001 is for CS = 1 representing High Priority Data (HPD). See Fig. 7. In order words, HPN is pre-configured with CS = 1, and LPN are pre-configured with CS = 0 before the commencement of network operations. In this way, it becomes impossible for any of the node types to behave greedily in favor of its class of data. As either of the data class traverses the network, intermediate or relay nodes computes the shortest traversal distance between source and destination node. Further details are presented in Sections 3.1 and 3.1.1. |
| 3. | If the packet delivery ratio for LPD is very low for 100 nodes (Fig. 9), what will happen to Fig. 13 if the number of nodes is increased to 100? The same argument does for Fig. 10, 11, 12 also. Thus, for Sec. 12, all simulation needs to be updated to at least 100 nodes. As the most practical network is much larger than this, it begs the question of the number of nodes is very large, then will the proposed PSPCM will lose its advantage? | Thank you for your comments and question.  The number of nodes has been increased from 50 to 200 for comparison with related mechanisms (i.e. ETX, QCOF, QWL-RPL, RPL-EC, RPL-FL) and the simulations/experimentations was reperformed, results was used to plot the graphs - Figs 16, 17, 18 and 19 for PDR, Power consumption, Throughput, and Convergence time respectively. The findings is supported by discussion in Section 5.2. Subsequently, to show support for network scalability of the proposed mechanism – PSPCM, and its HPD and LPD comparison, the number of simulated nodes was increased from 100 to 300, and the simulations/experimentations were reperformed. The obtained results were used to plot the graphs – Figs 12, 13, 14, and 15, for PDR, Power consumption, Throughput, and Convergence time respectively. A detailed discussion of the findings and is presented in Section 5.1. |
| 4. | As LPD packages will be less prioritized, then there is an issue of inability to guarantee Quality of Service (QoS) for these packages. How this can be resolved? | Thank you for your comments and question.  A much clearer explanation as to how PSPCM works have been presented in Section 3.1.6. The applications of the mechanism have also been presented in Figs. 3 and 4 (updated/improved) of Section 3.1. In this Figure, it is shown that High Priority Nodes (HPN), which generates HPD are installed in locations whose data are deemed critical, time-sensitive, and of high priority, likewise, Low Priority Nodes (LPN) which generates LPD are installed in locations whose data are less critical delay-tolerant and low priority. HPN and LPN are preconfigured and assigned a Class of Service (CS) of 1 and 0 respectively. This is implemented in the *Reserved* field of the modified DIO Base Object as presented in Fig 7 (updated) with explanations provided in Sections 3.1.1, while Section 3.1.6 presents the details of how the proposed PSPCM works. With this approach, data emanating from either HPN or LPN are routed to the sink using Eqs 14 and 16 for HPD or Eqs 15 and 17 for LPD. This approach helps to check and prevent any data from acting greedily to the detriment of the other data class, thereby preserving and improving the Quality of Service (QoS). |
| 5. | 5G wireless network system, which is upcoming, resolves a lot of existing issues with high priority data and latency. The authors need to compare their results with 5G protocols. | Thank you very much for your comments.  While 5G is gradually gaining the center-stage particularly for high-speed internet, with very minimal latency, at the moment, it will not completely wipe-out the existing technologies like 4G and WSN. One major drawback of 5G is the high cost of implementation and high power/energy consumption, which currently does not seem good for low-power and lossy network (LLN) devices. On the other hand, LLN devices have the advantage of very low power consumption making it possible for them to be installed in just any location and left unattended for several months or years without battery replacement. To make the best of both technologies, 5G and WSN can be implemented in conjunction together in a multi-tiered network, where the lower-tier is for WSN (data generation and aggregation) and the upper-tier is for transporting the aggregated data by high-speed 5G network for either immediate processing and or storage. This is what we implemented in our approach. Further detail of this is presented in Fig. 3, and Section 3.1. |

Again, thank you for giving us the opportunity to strengthen our manuscript with your valuable comments and queries. We have worked hard to incorporate your feedback and hope that these revisions persuade you to accept our submission.

Sincerely yours,

***Innocent Uzougbo ONWUEGBUZIE***

***(****Corresponding Author)*

*Universiti Teknologi Malaysia (UTM)*

*+601160770749*
