# Supplementary material for: Prioritized Shortest Path Computation Mechanism (PSPCM) for wireless sensor networks
Source: PLoS One. 2022 Mar 10;17(3):e0264683. doi: 10.1371/journal.pone.0264683 (PMC8912138; doi:10.1371/journal.pone.0264683)
Supplement: Attachment — Submitted filename: Responses to Reviewers - Rebuttal Letter - Latest Version.docx [file pone.0264683.s003.docx]

**Innocent Uzougbo ONWUEGBUZIE**
School of Computing, Faculty of Engineering,

Universiti Teknologi Malaysia (UTM)
Johor Bahru, Johor, Malaysia

2nd August 2021.

The Editor,

*PLoS ONE*

Dear Editor,

**Rebuttal Letter**

Thank you for inviting us to submit a revised version of our manuscript entitled, "***Prioritized shortest path computation mechanism (PSPCM) for wireless sensor networks***" for consideration by *PLoS ONE*. We also appreciate the time and efforts you and the reviewers have put in place to providing meticulous feedback on ways to improve our paper. This has offered us the opportunity to submit a revised version of our paper for your further consideration. We have taken time to implement all recommendations and changes suggested by the reviewers. We hope that the current revised version satisfactorily addresses all issues raised.

Our responses to reviewers comments is presented in a tabular format below:

**Responses to comments by Reviewer 3**

|  | **Reviewers’ Comments** | **Responses** |
| --- | --- | --- |
| 1. | The manuscript should be written in standard English, all grammar and typographical errors should be corrected | Thank you so much for your comments.  All grammatical and typographical errors in the article has been corrected. |
| 2. | The organization of the manuscript should be improved to highlight the objective, method, and main contribution | Thank you for your comments.  The organization of the article has been improved to highlight the objective, method and main contribution. This is presented in the 6^th^ paragraph of Section 1: Introduction. |
| 3. | The author is expected to demonstrate the main advantage of the current method. | Thank you for your comments.  The main advantage of the proposed mechanism is in its ability to take into consideration the heterogenous data nature of WSN, classifying them and generating efficient and optimal shortest routing paths leading to the destination node (sink) while ensuring that the integrity of the network is not compromised. This detail is presented in paragraph six (6) of Section 1: Introduction, and Section 6 of Conclusion and future work |
| 4. | Part 2 is to be improved, and it could be more concise. | Thank you for your comments.  Part 2 of the article has been improved with the review of recent literatures in a concise manner. |

Again, thank you for giving us the opportunity to improve the quality of our manuscript with your valuable comments and recommendations. We hope that this revised version is accepted for publication in your prestigious journal.

Sincerely yours,

***Innocent Uzougbo ONWUEGBUZIE***

***(****Corresponding Author)*

*Universiti Teknologi Malaysia (UTM)*

*+601160770749*
