# Supplementary material for: Prioritized Shortest Path Computation Mechanism (PSPCM) for wireless sensor networks
Source: PLoS One. 2022 Mar 10;17(3):e0264683. doi: 10.1371/journal.pone.0264683 (PMC8912138; doi:10.1371/journal.pone.0264683)
Supplement: Attachment — Submitted filename: Responses to Reviewers - Rebuttal Letter - Noveber Version.docx [file pone.0264683.s004.docx]

**Innocent Uzougbo ONWUEGBUZIE**
School of Computing, Faculty of Engineering,

Universiti Teknologi Malaysia (UTM)
Johor Bahru, Johor, Malaysia

30^th^ November 2021.

The Editor,

*PLoS ONE*

Dear Editor,

**Rebuttal Letter**

Thank you for inviting us to submit a revised version of our manuscript entitled, "***Prioritized shortest path computation mechanism (PSPCM) for wireless sensor networks***" for consideration by *PLoS ONE*. We also appreciate the time and efforts you and the reviewers have put in place to provide meticulous feedback on ways to improve our paper. This has offered us the opportunity to submit a revised version of our paper for your further consideration. We have taken time to implement all recommendations and changes suggested by the reviewer. We hope that the current revised version satisfactorily addresses all issues raised.

Our responses to the reviewer comments are presented in a tabular format below:

**Responses to comments by Reviewer 4**

|  | **Reviewer Comments** | **Responses** |
| --- | --- | --- |
| 1. | More achievements on this topic should be added for the Section "introductions" and "Related work" | Thank you for your comments and suggestions.  More achievements have been added to the “Introduction” and “Related work” sections, and their references have been included in the References section. |
| 2. | Mathematics modeling to analyze the method is not enough. | Thank you for your comments.  The mathematics modeling to analyze the proposed mechanism has been improved and presented in a more detailed manner. This is shown in Section 3.1.5 |
| 3. | Some references are out-of-date, so these references before 2014 should be deleted. At the same time, many important recent references are missing, which can support the idea of this paper, the following references should be added in the Section "References" | Thank you for your comments and suggestions.  All out-of-date references before 2014 have been removed and updated with up-to-date references as suggested. Please refer to the References section. |
| 4.. | The writing format of the paper should be revised. | Thank you for your comments and suggestions.  The writing format of this paper has been revised as suggested. |
| 5. | The format of some references in this paper does not completed. | Thank you for your comments.  References with incomplete format have been completely removed and updated with properly formatted and up-to-date references. Kindly refer to the References section. |

Again, thank you for giving us the opportunity to improve the quality of our manuscript with your valuable comments and recommendations. We hope that this revised version is accepted for publication in your prestigious journal.

Sincerely yours,

***Innocent Uzougbo ONWUEGBUZIE***

***(****Corresponding Author)*

*Universiti Teknologi Malaysia (UTM)*

*+601160770749*
